# Impact of combined ginsenoside‐MC1 and irisin on mitochondrial apoptosis in diabetic rats with hepatic reperfusion injury: Role of AMPK/JNK signalling

**DOI:** 10.1113/EP092982

**Published:** 2025-09-10

**Authors:** Jie Lin, Lei Han, Zhigang Ma, Bo Yuan, Yabin Yu

**Affiliations:** ^1^ Organ Transplantation Center The First Affiliated Hospital of Kunming Medical University Kunming Yunnan China; ^2^ Department of Hepatobiliary Surgery The Affiliated Huaian No.1 People's Hospital of Nanjing Medical University Huaian Jiangsu China

**Keywords:** apoptosis, ginsenosides, hepatic ischaemia–reperfusion injury, irisin, mitochondria, type 2 diabetes mellitus

## Abstract

Hepatic ischaemia–reperfusion (IR) injury is a serious clinical issue, especially in patients with type 2 diabetes mellitus (T2DM). As mitochondria play a critical role in the regulation of IR‐induced liver damage, mitochondria‐targeted treatment is of the utmost significance for improving outcomes. The present study explored the mitoprotective role of combined ginsenoside‐MC1 (GMC1) and irisin administration in diabetic rats with hepatic IR injury. T2DM was induced in male Sprague–Dawley rats with a high‐fat diet and a low‐dose streptozotocin. Following the induction of diabetes, hepatic IR injury was induced. Rats were pretreated with GMC1 and/or irisin for 28 days prior to IR injury. Liver function was evaluated by quantitation of serum alanine aminotransferase (ALT), aspartate aminotransferase (AST) and lactate dehydrogenase (LDH). Histopathological changes were observed with haematoxylin–eosin staining. Apoptotic markers (Bax, Bcl‐2, cleaved caspase‐3) and signalling proteins (AMP‐activated protein kinase (AMPK), c‐Jun N‐terminal kinase (JNK)) were examined by western blotting. Mitochondrial function was evaluated by measuring reactive oxygen species, membrane potential and ATP content. Oxidative stress markers, such as malondialdehyde (MDA), superoxide dismutase (SOD), and glutathione peroxidase (GPx), were also measured. Combined therapy lowered AST, ALT and LDH levels, and histopathological injury (*P *< 0.05). It restored mitochondrial function; upregulated Bcl‐2 and phosphorylated AMPK expression; downregulated Bax, cleaved caspase‐3 and phosphorylated JNK expression; and reduced MDA levels, while elevating SOD and GPx activity (*P *< 0.05). AMPK inhibition by compound C reversed these protective effects. GMC1–irisin combination therapy safeguarded diabetic rats against IR‐caused liver damage through suppressing mitochondrial apoptosis by AMPK/JNK signalling, a hopeful therapeutic approach in diabetic patients.

## Abstract (highlights)

- **What is the central question of this study?**
  Can combined ginsenoside‐MC1 and irisin administration mitigate hepatic ischaemia–reperfusion injury in type 2 diabetic rats by targeting mitochondrial apoptosis via AMP‐activated protein kinase (AMPK)/c‐Jun N‐terminal kinase (JNK) signalling?
- **What is the main finding and its importance?**
  Combined therapy reduced ALT, AST and LDH release, oxidative stress and apoptotic markers, restoring mitochondrial function via AMPK activation and JNK suppression, representing a promising mitochondria‐targeted strategy to improve outcomes in diabetic patients undergoing hepatic IR injury.

## 1 INTRODUCTION

Ischaemia–reperfusion (IR) injury in the liver is a major clinical challenge, particularly during liver transplantation, resection surgery, trauma and shock. It is estimated to contribute to up to 25% of early post‐transplant complications (Guo et al., 2022). Hepatic IR involves a temporary interruption of blood flow followed by reperfusion, which leads to ATP depletion, oxidative stress, mitochondrial dysfunction, inflammation and apoptosis (Nakazato et al., 2018). If not effectively managed, it can result in significant liver dysfunction, systemic inflammation, and multi‐organ failure, highlighting the need for effective therapeutic strategies (George et al., 2024). The presence of type 2 diabetes mellitus (T2DM) also enhances hepatic IR damage, enhancing susceptibility to cellular damage and diminishing the efficacy of any prospective therapeutic measures. Diabetes is represented by hyperglycaemia, oxidative stress, low‐grade inflammation and mitochondrial dysfunction, which all enhance the harmful effect of IR damage to the liver. In addition, diabetic conditions cause endothelial dysfunction, enhanced production of pro‐inflammatory cytokines, and reduced antioxidant defence, rendering the liver more susceptible to ischaemic injury (Behrends et al., 2010; Rao et al., 2017; Y. Zhang et al., 2016; Zhijun et al., 2023). Furthermore, in diabetic conditions, the breakdown of adaptive cytoprotective mechanisms also reduces the efficacy of traditional therapies (Mohamed et al., 2016; Shi et al., 2020). With the worldwide increase in the prevalence of diabetes and its correlation with hepatic dysfunction, the need to develop therapy interventions that will counteract hepatic IR damage in diabetic patients is an urgent clinical and research imperative.

Mitochondria are key modulators of hepatic IR injury and are targets and mediators of cell injury (Yang et al., 2018). Mitochondrial dysfunction in IR situations is defined by decreased ATP content, overproduction of reactive oxygen species (ROS), loss of mitochondrial membrane potential, and activation of mitochondrial apoptotic pathways. This results in cytochrome *c* release, caspase activation and hepatocyte apoptosis, aggravating hepatic injury (S. Zhang, Rao, Yang, et al., 2022). Of the critical upstream regulatory cascades that modulate mitochondrial homeostasis, the AMP‐activated protein kinase (AMPK)/c‐Jun N‐terminal kinase (JNK) signalling cascade is a critical regulator of hepatocyte survival or cell death (Chen et al., 2018). AMPK, the energy master sensor, enhances mitochondrial integrity, alleviates oxidative stress, and increases autophagic removal of damaged organelles, thus having a protective effect against IR injury (Cai et al., 2022). Conversely, JNK activation is linked to heightened mitochondrial permeability, ROS generation and cell death, and its regulation is therefore the optimal target for therapy (King et al., 2009). Notably, in diabetic conditions, AMPK activation is suppressed, while JNK activity is exacerbated, further enhancing mitochondrial dysfunction and apoptotic responses in hepatic IR injury (Entezari et al., 2022). Therefore, targeting the mitochondrial apoptotic pathway via modulation of AMPK/JNK signalling represents a promising strategy to reduce hepatic IR‐induced damage, particularly in diabetic settings (Paskeh et al., 2022; Yang et al., 2018).

Despite extensive research on hepatoprotective interventions, monotherapy approaches have largely failed to translate into clinical success, particularly in the presence of comorbidities such as diabetes (Kong et al., 2009; D.‐Y. Li et al., 2018; Patel & Saravolatz, 2006). Given the complex and multifactorial nature of hepatic IR injury, a single‐agent approach may be insufficient to provide optimal protection. Combination therapies, by simultaneously targeting multiple pathological pathways, hold greater potential to achieve additive or synergistic protective effects, thereby improving treatment efficacy (Mao et al., 2022; Marubayashi et al., 2000). In this context, ginsenoside‐MC1 (GMC1) and irisin represent two promising bioactive compounds with distinct yet complementary mechanisms of action. GMC1, a pharmacologically active ginseng constituent, has shown strong antioxidant, anti‐inflammatory and anti‐apoptotic effects in multiple models of disease (Roh et al., 2020). It has been shown to modulate mitochondrial function, reduce ROS generation, and increase cellular resilience against IR damage (M. Wang & Li, 2022). GMC1 stimulates AMPK signalling and thus induces mitochondrial biogenesis and suppresses apoptosis (Hong et al., 2020; H. Li et al., 2024). GMC1 has also been proposed in hepatic IR injury to suppress oxidative stress and to enhance hepatocyte survival and is a potential candidate to be developed as a therapy (Bian et al., 2023; Hao et al., 2025; Wang, Zhu, Jia, et al., 2022). Likewise, irisin, an exercise‐myokine variant derived from fibronectin type III domain‐containing protein 5 (FNDC5), has gained attention for its metabolic and cell protection properties. Irisin has been reported to increase mitochondrial biogenesis and energy metabolism, suppress inflammation and inhibit apoptotic signalling in tissues such as the liver (Bi et al., 2019; Zhao et al., 2022). Studies have demonstrated that irisin induces AMPK activation and suppresses JNK‐mediated apoptosis following intracerebral haemorrhage in mice (Y. Wang, Tian, Tan, et al., 2022). In addition, irisin is anti‐diabetic and insulin‐sensitizing, which is of special significance in the case of diabetes‐related hepatic injury (Liu et al., 2015).

Given the multi‐factorial nature of hepatic IR injury and the existence of different overlapping pathological pathways, combination therapy may become a requirement for offering better hepatoprotection (Teoh, 2011). For this purpose, GMC1 and irisin, by targeting either distinct or converging signalling pathways, may have the potential to generate synergistic protective effects against hepatic IR injury under diabetic conditions. Consistent with this, the present work is designed to explore the hepatoprotective effects of GMC1 and irisin against IR injury in diabetic rats involving mitochondrial apoptosis, AMPK/JNK signalling pathways and regulation of oxidative stress. By disclosing the underlying mechanisms, the current work presents new evidence of prospective therapeutic targets of hepatic IR injury in diabetes patients, acting to fill a crucial translational gap in research into hepatoprotection.

## 2 METHODS

### 2.1 Ethical approval

All experimental procedures were approved by the Kunming Medical University Animal Experiment Ethics Review Committee, China (Ethics Code: kmmu20221523). The study was conducted in strict accordance with the guidelines outlined in the 8th Edition (2011 revision) of the National Institutes of Health Guide for the Care and Use of Laboratory Animals, with full adherence to the ARRIVE 2.0 standards. Animal welfare and efforts to minimize suffering were prioritized throughout the study. Euthanasia was performed according to the AVMA Guidelines for the Euthanasia of Animals.

### 2.2 Experimental animals

A total of 66 male Sprague–Dawley rats, aged 8–10 weeks and weighing 200–250 g, were obtained from the Laboratory Animal Centre of Kunming Medical University, China. Animals were housed under controlled conditions with temperature maintained at 20–24°C, relative humidity at 55%, and a 12‐h light–dark cycle. They had ad libitum access to standard chow and water. Six rats were maintained on a regular diet as non‐diabetic controls, while the other 60 rats were induced with T2DM for 12 weeks. Non‐diabetic animals served as controls for evaluating biochemical changes with respect to T2DM and confirmation of successful model development of diabetes. Upon establishment of diabetes, diabetic rats were allocated into five groups on the basis of Experimental Phases 1 and 2, and underwent hepatic IR damage with or without drug preconditioning.

### 2.3 Development of the experimental T2DM model

High‐fat diet (HFD) and low‐dose streptozotocin (STZ) treatment were used to induce T2DM in rats. The animals initially had a 2‐week adaptation phase before diabetic induction. Then they were kept on a specially prepared diet that consisted of normal chow supplemented with 0.05% cholate, 1% cholesterol and 8% lard (w/w). The altered diet allowed the total caloric diet to have around 62% of its calories as fat. In week 7, following 8 h of overnight fasting, rats were given an intraperitoneal injection of STZ (35 mg/kg; Sigma‐Aldrich, St Louis, MO, USA) in a pH 4.5 citrate buffer solution. Three days later, blood was drawn from the tail vein, and the fasting blood sugar (FBS) content was measured using a glucometer (Roche, Indianapolis, IN, USA). Rats with FBS levels exceeding 250 mg/dL were considered diabetic and included in the study, while seven rats that did not meet this criterion were excluded from further experimentation. To ensure the stability of the diabetic state, the rats continued on the high‐fat diet for an additional 6 weeks, bringing the total diabetes induction period to 12 weeks. Body weights were recorded on a weekly basis, and after confirming diabetes, the rats were randomly assigned to experimental groups, as outlined in the following section.

### 2.4 Experimental design and group allocation

This study was conducted in two distinct experimental phases, each focusing on different aspects of hepatoprotection in diabetic rats.

#### 2.4.1 First experimental phase (*n* = 30)

The primary objective of this phase was to evaluate the relative effectiveness of the combination therapy compared to each monotherapy in alleviating hepatic IR injury under T2DM conditions. Key parameters for evaluating liver function included serum levels of aspartate aminotransferase (AST), alanine aminotransferase (ALT) and lactate dehydrogenase (LDH), as well as histopathological assessments conducted 24 h post‐reperfusion. The diabetic rats were randomly assigned to five arms (*n* = 6 per group):

1. Sham group: Underwent laparotomy surgery without hepatic IR induction.
2. IR group: Subjected to 30 min of hepatic ischaemia, followed by 24 h of reperfusion.
3. IR + GMC1 group: Received GMC1 (10 mg/kg/day, intraperitoneally; dissolved in sterile saline; Ambo Institute, Seoul, Korea) once daily for 28 consecutive days prior to induction of IR injury (Yanwei Zhang, Xu, & Yanqing Zhang, 2022).
4. IR + Irisin group: Received recombinant rat irisin (0.5 mg/kg/day, intraperitoneally; reconstituted in phosphate‐buffered saline; Cloud‐Clone, China; cat. no.: APN576Mu01) once daily for 28 consecutive days prior to IR induction (Xu et al., 2022).
5. IR + GMC1 + Irisin group: Treated with both GMC1 and irisin, as described in groups 3 and 4.

#### 2.4.2 Second experimental phase (*n* = 30)

The second phase was designed to elucidate the molecular mechanisms underlying the hepatoprotective effects of combination therapy. Since findings from the first phase indicated superior protection with the combined approach, monotherapy groups were excluded in this phase. Instead, the study focused on analysing key molecular pathways implicated in hepatic IR injury. To specifically examine the role of the AMPK signalling pathway, the AMPK inhibitor compound C (CC) was introduced to the combination therapy group. After 24 h of reperfusion, liver function was assessed by measuring serum levels of AST, ALT and LDH, along with histopathological evaluation. Additionally, liver tissues were collected under deep anaesthesia for biochemical and molecular analyses. The diabetic groups (*n* = 6 per group) were structured as follows:

1. Sham group: Subjected to laparotomy without IR induction.
2. IR group: Underwent 30 min of ischaemia, followed by 24 h of reperfusion.
3. IR + CC group: Treated with compound C (0.2 mg/kg, intravenously, every 72 h; dissolved in 10% DMSO diluted with sterile saline; Sigma‐Aldrich) for 28 days prior to IR induction to inhibit AMPK activity (Han et al., 2023).
4. IR + GMC1 + Irisin group: Received combination therapy (GMC1 + Irisin) as outlined in the first phase.
5. IR + CC + GMC1 + Irisin group: Administered compound C alongside GMC1 and irisin to investigate the effect of AMPK inhibition on hepatoprotection.

At 24 h post‐reperfusion in both experimental phases, animals were deeply anaesthetized via intraperitoneal injection of ketamine (60 mg/kg) and xylazine (10 mg/kg). Blood samples were collected from the postcaval vein for serum analysis of liver function markers. Following blood collection, euthanasia was performed by thoracotomy in accordance with the AVMA Guidelines for the Euthanasia of Animals. Liver tissues were then harvested; in the first experimental phase, samples were primarily used for histopathological assessment, while in the second phase they were further processed for molecular and signalling pathway analyses.

### 2.5 Evaluation of metabolic alterations in T2DM

To assess glycaemic control, FBS levels were measured using a glucometer (Convergent Technologies, Cölbe, Germany). Plasma insulin concentrations were quantified using an enzyme‐linked immunosorbent assay (ELISA) kit (Rat Insulin ELISA Kit, cat. no. EZRMI‐13K, Millipore, Billerica, MA, USA), following the manufacturer's recommendations. The degree of insulin resistance was determined through the homeostasis model assessment of insulin resistance (HOMA‐IR), which was calculated using the following equation: HOMA‐IR = [Fasting insulin (µU/mL) × Fasting glucose (mmol/L)]/22.5. For lipid metabolism evaluation, total cholesterol levels were analysed using a cholesterol oxidase–peroxidase (CHOD‐PAP) enzymatic colorimetric assay, employing the Rat Cholesterol Assay Kit (cat. no. 10007640, Cayman Chemical Co., Ann Arbor, MI, USA), as per the manufacturer's protocol.

### 2.6 Surgical procedure for hepatic IR model

To prepare for the surgical procedure, rats underwent an overnight fasting period before being anaesthetized via intraperitoneal injection of ketamine (60 mg/kg) and xylazine (10 mg/kg). Once fully anaesthetized, they were placed in a supine position on a pre‐sterilized surgical platform and immobilized to ensure procedural stability. As spontaneous breathing remained adequate and airway obstruction was not expected, endotracheal intubation was not required. A ∼2 cm longitudinal incision was carefully made along the upper abdominal midline, followed by gradual dissection of the abdominal layers to expose the liver. The peri‐hepatic ligament was gently dissected to reveal the hepatoduodenal ligament, facilitating access to the hepatic vasculature. To induce hepatic ischaemia, the Pringle manoeuvre was applied using a non‐traumatic vascular clamp, temporarily obstructing the portal vein, hepatic artery and bile duct (Frich et al., 2006). The ischaemic phase was maintained for 30 min, after which the clamp was released to allow reperfusion for 24 h. The incision site was then carefully sutured in layers to ensure proper wound closure. For the sham group, all surgical steps were identical, except that vascular occlusion was not performed, ensuring normal hepatic blood flow was maintained throughout the procedure. Upon full recovery from anaesthesia, rats were monitored and then returned to their cages with unrestricted access to food and water. Buprenorphine (0.05 mg/kg, subcutaneously) was administered immediately post‐surgery and continued every 12 h for 24 h to provide analgesia.

### 2.7 Biochemical assessment of liver function and oxidative stress markers

Blood samples were collected from the postcaval vein, then centrifuged at 3000 *g* for 10 min to separate the serum, which was stored at −20°C for further analysis. Serum levels of AST, ALT and LDH were quantified using an automated biochemical analyser (Hitachi, Tokyo, Japan). For tissue analysis, liver specimens were carefully excised from the median lobe, rinsed with cold saline to remove any debris, and immediately immersed in liquid nitrogen at −196°C to preserve their biochemical integrity. The samples were then kept at −80°C until analysis. Frozen liver samples were thawed at 4°C before biochemical analysis, then homogenized in an ice‐cold phosphate buffer (pH 7.4). Homogenates were centrifuged at 3500 *g* for 10 min at 4°C, and supernatants were utilized to assay markers of oxidative stress, which included malondialdehyde (MDA), superoxide dismutase (SOD) and glutathione peroxidase (GPx). These biomarkers were quantified by spectrophotometer at 532 nm for MDA and 460 nm for SOD and GPx. Protein content in the liver homogenates was determined by colorimetric assay kits (Jiancheng Bioengineering, Nanjing, China). The MDA, SOD and GPx results are presented in picograms per milligram of protein in the liver samples.

### 2.8 Microscopic evaluation of liver histopathology

Liver tissue samples were obtained carefully and immediately fixed in 4% paraformaldehyde to ensure structural integrity. Following fixation, the samples were dehydrated, embedded in paraffin, and cut into 4‐µm sections for histological analysis. Sections were stained with haematoxylin–eosin (H&E) to permit detailed examination of cellular and tissue morphology. Histopathological evaluation was performed using a light microscope (Olympus CH30, Olympus, Tokyo, Japan) at a magnification of ×400. Liver injury severity was determined based on the Suzuki grading system, which classifies histological alterations on a 0–4 scale. A score of 0 indicated a normal liver with no signs of necrosis, sinusoidal congestion or cellular vacuolization. Scores of 1–3 correspond to increasing levels of vascular congestion and hepatocellular degeneration, while a score of 4 reflects extensive necrosis affecting more than 60% of the liver tissue. To facilitate statistical analysis, the cumulative Suzuki score was utilized as a quantitative measure of liver damage.

### 2.9 Protein expression analysis via western blot

To investigate protein expression levels, liver tissues (30 µg) were homogenized in a lysis buffer supplemented with protease and phosphatase inhibitors and centrifuged at 12,000 *g* for 15 min at 4°C, yielding total protein extracts. The extracted proteins were separated using SDS‐PAGE, where 10% gels were utilized for proteins ≥60 kDa, and 12% gels for those <60 kDa. After electrophoresis, proteins were transferred onto polyvinylidene fluoride (PVDF) membranes for subsequent immunoblotting. To block non‐specific binding, the membranes were incubated in 5% non‐fat dry milk prepared in Tris‐buffered saline containing 0.1% Tween‐20 (TBST) at room temperature for 1 h. The membranes were then incubated overnight at 4°C with specific primary antibodies, including Bcl‐2‐associated X protein (Bax) (1:1000; Cell Signaling Technology, Danvers, MA, USA), B‐cell lymphoma 2 (Bcl‐2) (1:500; Cell Signaling Technology), cleaved caspase‐3 (1:1000; Cell Signaling Technology), total caspase‐3 (1:1000; Cell Signaling Technology), phosphorylated AMPKα (p‐AMPK; Thr172; 1:500; Cell Signaling Technology), total AMPK (1:500; Cell Signaling Technology), phosphorylated JNK (p‐JNK; Thr183/Tyr185; 1:1000; Cell Signaling Technology), total JNK (1:1000; Cell Signaling Technology), and glyceraldehyde‐3‐phosphate dehydrogenase (GAPDH) (1:5000; Cell Signaling Technology). After primary incubation, membranes underwent multiple washes with TBST before being treated with a horseradish peroxidase (HRP)‐conjugated goat anti‐rabbit secondary antibody (1:5000; Beyotime, Shanghai, China) at room temperature for 1 h. Protein detection was performed using an enhanced chemiluminescence (ECL) kit (Bio‐Rad Laboratories, Hercules, CA, USA), and protein band intensities were analysed with ImageJ software. Protein expression values were normalized against GAPDH, which served as an internal control.

### 2.10 Procedure of mitochondria isolation

Freshly isolated liver mitochondria were obtained using a differential centrifugation method. Briefly, liver tissue was excised under sterile conditions and immediately placed in ice‐cold isolation buffer (220 mM mannitol, 70 mM sucrose, 10 mM HEPES, 1 mM EGTA, pH 7.4). The tissue was finely minced and homogenized using a glass–Teflon homogenizer (10 strokes) on ice. The homogenate was centrifuged at 800 *g* for 10 min at 4°C to remove nuclei and cellular debris. The supernatant was collected and centrifuged again at 10,000 *g* for 15 min at 4°C to pellet mitochondria. The mitochondrial pellet was washed once in isolation buffer and resuspended in a minimal volume of ice‐cold buffer for immediate use in downstream assays. Protein concentration was determined using a Bradford assay kit (Bio‐Rad).

### 2.11 Measurement of mitochondrial ROS production

Mitochondrial ROS levels were assessed using 2′,7′‐dichlorodihydrofluorescein diacetate (DCFDA) dye (Abcam, Waltham, MA, USA). Freshly isolated mitochondria (50 µg protein) were incubated in Hank's balanced salt solution (HBSS) buffer supplemented with 5 µM DCFDA at 37°C for 30 min in the dark. Fluorescence intensity was measured using a microplate reader (excitation: 485 nm, emission: 535 nm). Background fluorescence was subtracted from all readings, and ROS levels were normalized to protein content.

### 2.12 Assessment of mitochondrial membrane potential

Mitochondrial membrane potential was determined using the JC‐10 assay kit (Sigma‐Aldrich), which exhibits potential‐dependent accumulation within mitochondria, shifting fluorescence emission from green (∼525 nm) to red (∼590 nm) in healthy mitochondria. Isolated mitochondria (50 µg protein) were incubated with 5 µM JC‐10 dye in mitochondrial assay buffer at 37°C for 20 min. Fluorescence intensity was measured using a fluorescence plate reader, and the red/green fluorescence ratio was calculated to assess changes in mitochondrial polarization. A decrease in this ratio indicated mitochondrial depolarization. As a positive control for depolarization, an uncoupler agent, carbonyl cyanide *m*‐chlorophenyl hydrazone (CCCP, 50 µM), was used to validate the assay's sensitivity in detecting mitochondrial membrane potential loss.

### 2.13 Measurement of mitochondrial ATP production

ATP levels in isolated mitochondria were measured using a luciferase‐based ATP assay kit (Cayman Chemical Co.). Freshly prepared mitochondria (50 µg protein) were incubated in ATP assay buffer containing luciferin–luciferase reagent for 10 min at room temperature. Luminescence was recorded using a microplate luminometer, and ATP concentration was determined using a standard curve generated from known ATP concentrations. ATP levels were expressed as nanomoles of ATP per milligram of protein.

### 2.14 Statistical analysis

All statistical analyses were conducted using GraphPad Prism (version 9.5.1, GraphPad Software, Boston, MA, USA) for Windows. Continuous data are expressed as means ± standard deviation (SD). For comparisons between two groups, an independent Student's *t*‐test was performed. When comparing multiple groups, one‐way analysis of variance (ANOVA) followed by Tukey's *post hoc* test was employed to identify significant differences between groups. A *P*‐value of <0.05 was considered statistically significant.

## 3 RESULTS

### 3.1 Validation of the diabetic model through metabolic parameters

**FIGURE 1:**
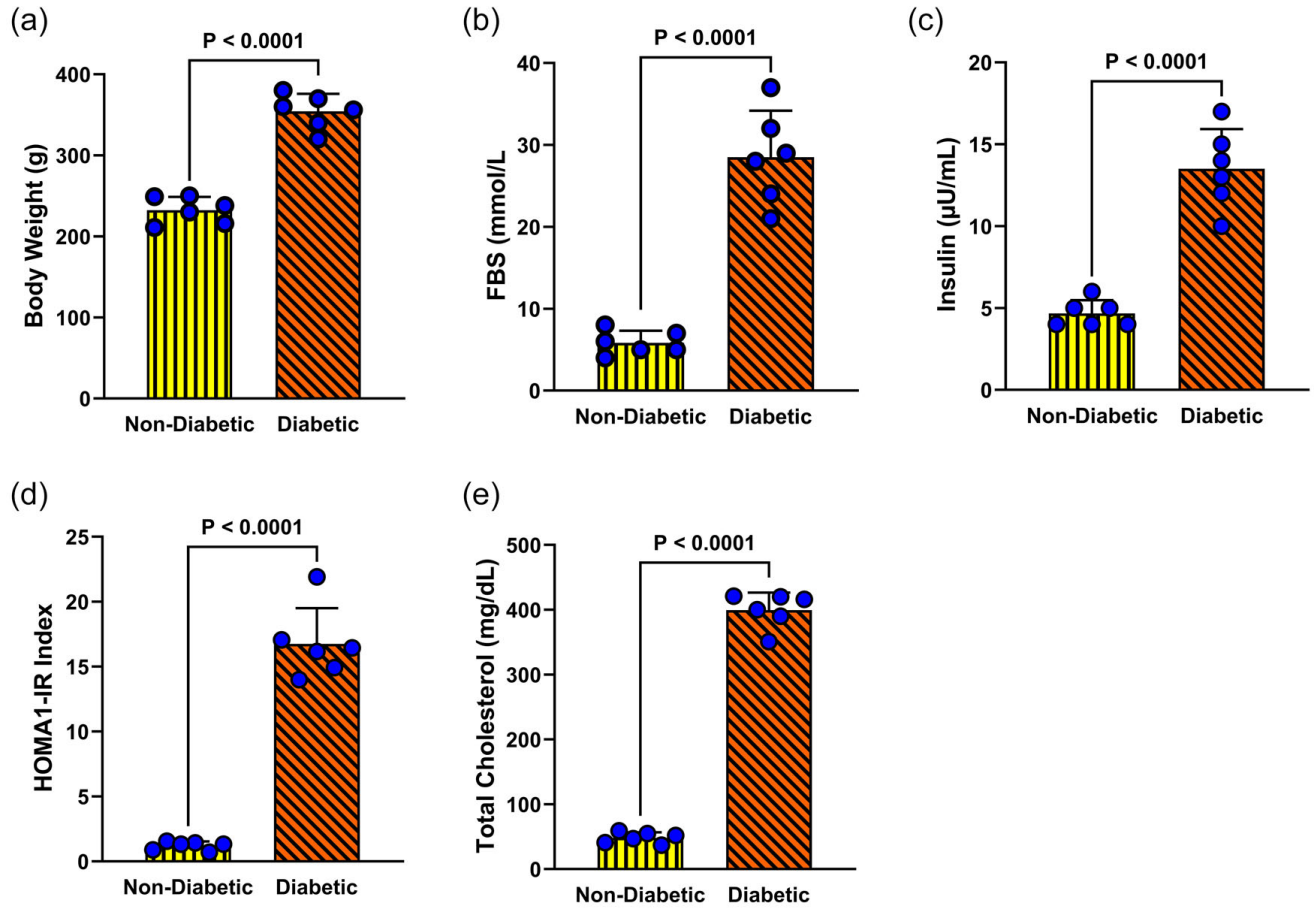
Metabolic characterization of the diabetic model. Key metabolic parameters were assessed to validate the induction of diabetes. Body weight (a), fasting blood sugar (FBS) (b), plasma insulin levels (c), insulin resistance index (HOMA1‐IR) (d), and total plasma cholesterol (e) were measured in diabetic and non‐diabetic rats. Statistical analysis was performed using Student's *t*‐test, with data presented as means ± SD. Each group consisted of six animals, with representative data from the diabetic group displayed in these graphs.

To confirm the successful establishment of the diabetic model, key metabolic parameters, including body weight, FBS, circulating insulin levels, insulin resistance index (HOMA‐1IR) and total cholesterol, were evaluated. At the end of the experimental period, Student's *t*‐test revealed a substantial increase in body weight (*P* < 0.0001), severe hyperglycaemia (*P* < 0.0001), elevated insulin levels (*P* < 0.0001), a significantly higher HOMA‐IR index (*P* < 0.0001), and a pronounced rise in total cholesterol (*P* < 0.0001) in diabetic rats compared to controls (Figure 1a–e). These profound metabolic disturbances confirm the successful development of diabetes, characterized by impaired glucose homeostasis, insulin resistance and dyslipidaemia.

### 3.2 Effects of monotherapies and combination therapy on liver function in diabetic rats with IR injury

**FIGURE 2:**
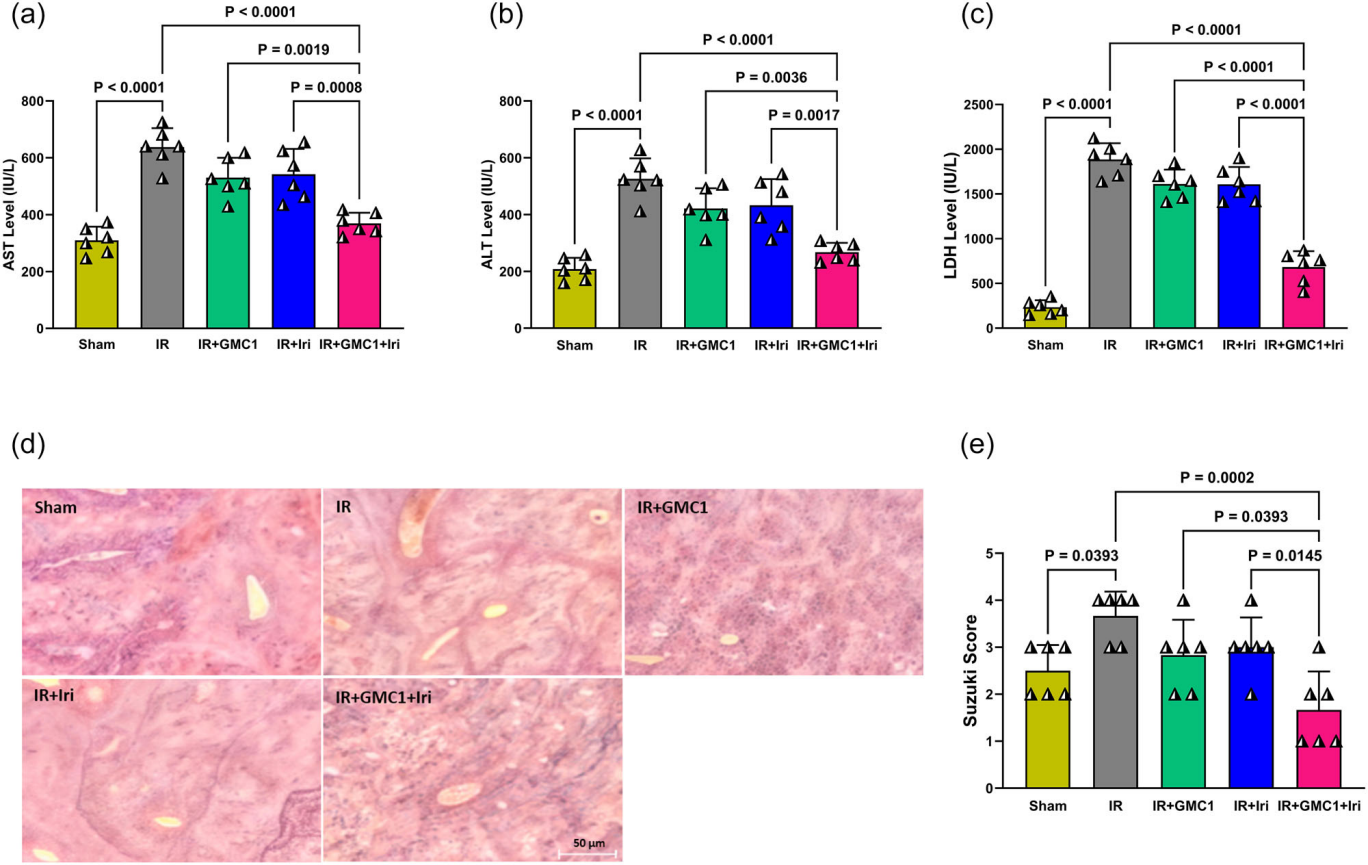
Effects of monotherapies and combination therapy on liver function in diabetic rats with IR injury. Serum levels of aspartate aminotransferase (AST) (a), alanine aminotransferase (ALT) (b), and lactate dehydrogenase (LDH) (c) were assessed as markers of liver injury. Representative H&E‐stained liver sections (d, magnification ×400, scale bar = 50 µm) illustrate histopathological changes across groups, while Suzuki's histological injury scores (e) quantify liver damage. Data are presented as means ± SD and analysed using one‐way ANOVA followed by Tukey's *post hoc* test (*n* = 6 per group). GMC1, ginsenoside‐MC1; IR, ischaemia–reperfusion; Iri, irisin.

As shown in Figure 2a–c, the IR group exhibited a significant increase in AST, ALT and LDH levels compared to the Sham group (*P* < 0.0001 for all), indicating severe hepatic injury. Monotherapies with GMC1 or irisin alone did not result in a significant reduction in these enzyme levels. However, pretreatment with both GMC1 and irisin in diabetic IR rats led to a substantial decrease in AST, ALT and LDH levels compared to the IR group (*P* < 0.0001 for all), suggesting an additive hepatoprotective effect (Figure 2a–c). Furthermore, the combination therapy demonstrated superior efficacy in reducing liver enzyme levels compared to either GMC1 or irisin alone (*P* < 0.01 to *P* < 0.0001, Figure 2a–c), highlighting its enhanced protective effects. Histological analysis (Figure 2d,e) further supported these findings. The IR group exhibited severe hepatic injury, characterized by hepatocyte vacuolization, disruption of lobular architecture, and sinusoidal congestion. While monotherapy‐treated groups showed partial improvement, the combination therapy group displayed the most pronounced hepatoprotection, with markedly preserved liver architecture (Figure 2d). According to Suzuki's histological classification based on H&E staining, the injury score in the IR group was significantly higher than that in the Sham group (*P* = 0.0393). Moreover, the injury score in the combination therapy group was significantly lower than in the IR group (*P* = 0.0002), the GMC1 group (*P* = 0.0393) and the irisin group (*P* = 0.0145), reinforcing the superior efficacy of dual therapy in mitigating hepatic damage (Figure 2e).

### 3.3 AMPK inhibition impairs the hepatoprotective effects of combination therapy in diabetic rats with IR injury

**FIGURE 3:**
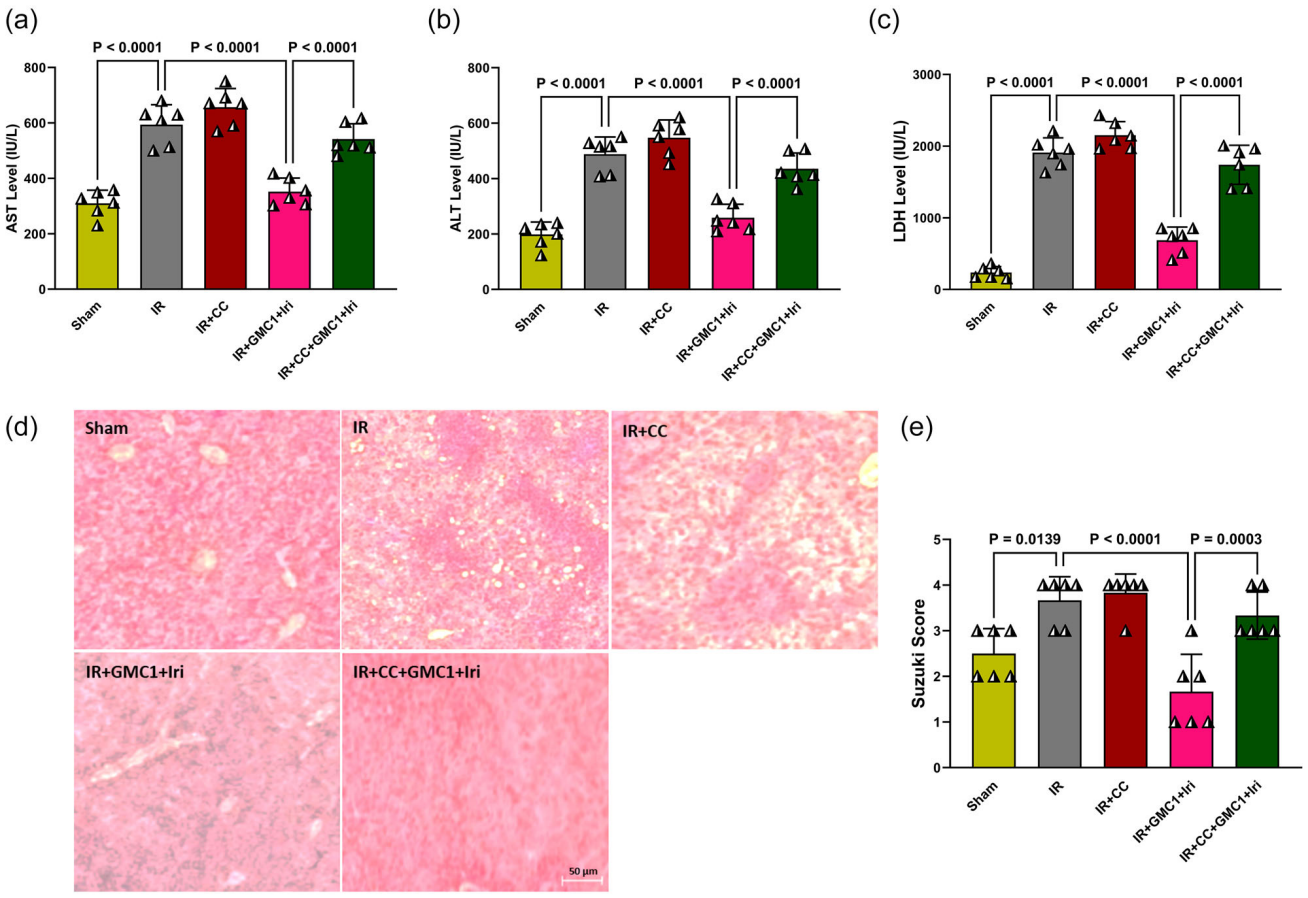
AMPK inhibition impairs the hepatoprotective effects of combination therapy in diabetic rats with IR injury. Serum levels of aspartate aminotransferase (AST) (a), alanine aminotransferase (ALT) (b), and lactate dehydrogenase (LDH) (c) were measured to evaluate liver injury. Representative H&E‐stained liver sections (d, magnification ×400, scale bar = 50 µm) depict histopathological alterations across groups, while Suzuki's histological injury scores (e) quantify liver damage severity. Data are presented as means ± SD and analysed using one‐way ANOVA followed by Tukey's *post hoc* test (*n* = 6 per group). CC, compound C; GMC1, ginsenoside‐MC1; IR, ischaemia–reperfusion; Iri, irisin.

Building on the previous protocol, which demonstrated the superior cardioprotective effects of combination therapy compared to monotherapies, we aimed to investigate the underlying mechanisms driving this protection. Specifically, we focused on examining the role of AMPK in the hepatoprotective effects of combination therapy by introducing an AMPK inhibitor (CC) exclusively within the combination therapy group. As shown in Figure 3a–c, induction of IR significantly elevated AST, ALT and LDH levels in the IR group compared to the Sham group (*P* < 0.0001 for all), indicating severe hepatic injury. In contrast, combination therapy with GMC1 and irisin significantly reduced AST, ALT and LDH levels (*P* < 0.0001 for all), highlighting the hepatoprotective potential of this treatment. However, when CC was administered as a pretreatment, it effectively counteracted the hepatoprotective effects of combination therapy, with a significant increase in AST, ALT and LDH levels similar to those observed in the IR group (*P* < 0.0001 for all) (Figure 3a–c). Histological analysis (Figure 3d,e) further corroborated these findings. Both the IR and IR + CC groups displayed severe hepatic damage, characterized by hepatocyte vacuolization, disruption of lobular architecture, and sinusoidal congestion. In contrast, the combination therapy group exhibited markedly preserved liver architecture and minimal injury. However, pretreatment with CC led to a significant reversal of these beneficial histological changes, with hepatic architecture resembling that of the IR group (Figure 3d). According to Suzuki's histological classification based on H&E staining, the injury score in the IR group was significantly higher than in the Sham group (*P* = 0.0139). The combination therapy group showed a significantly lower injury score compared to the IR group (*P* < 0.0001). However, pretreatment with CC significantly reversed the protective effects of combination therapy, as evidenced by a significantly higher injury score in the IR + CC + GMC1 + Iri group compared to the combination therapy group (*P* = 0.0003) (Figure 3e). These results underscore the importance of AMPK activation in mediating the hepatoprotective effects of combination therapy.

### 3.4 Combination therapy reduces apoptosis through the AMPK/JNK signalling pathway in diabetic IR‐injured livers

**FIGURE 4:**
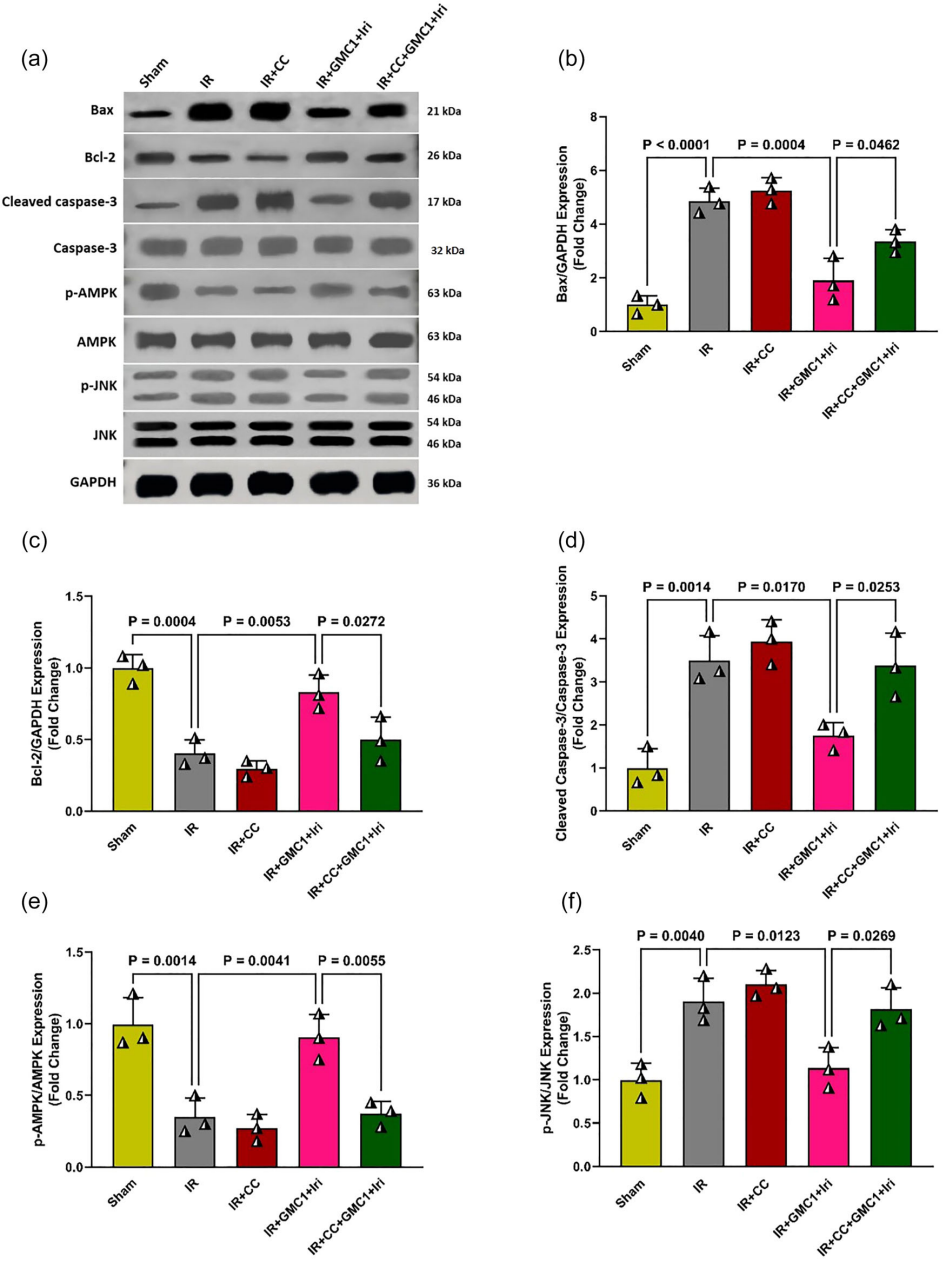
Combination therapy reduces apoptosis through the AMPK/JNK signalling pathway in diabetic IR‐injured livers. Panels show representative western blot images (a) and quantification of protein expression levels for Bcl‐2‐associated X protein (Bax) (b), B‐cell lymphoma 2 (Bcl‐2) (c), cleaved caspase‐3 (d), phosphorylated AMP‐activated protein kinase (p‐AMPK) (e), and phosphorylated c‐Jun N‐terminal kinase (p‐JNK) (f). Data are presented as means ± SD and analysed using one‐way ANOVA followed by Tukey's *post hoc* test (*n* = 6 per group). CC, compound C; GAPDH, glyceraldehyde‐3‐phosphate dehydrogenase; GMC1, ginsenoside‐MC1; IR, ischaemia–reperfusion; Iri, irisin.

To investigate the mechanisms underlying the hepatoprotective effects of combination therapy, the expression of key apoptotic and signalling proteins (Bax, Bcl‐2, cleaved caspase‐3, p‐AMPK and p‐JNK) was assessed in the livers of diabetic rats using western blot analysis (Figure 4a–f). In the IR group, a significant increase in Bax, cleaved caspase‐3 and p‐JNK protein levels was observed compared to the Sham group (*P* < 0.0001, *P* = 0.0014 and *P* = 0.0040, respectively), while there was a significant decrease in Bcl‐2 and p‐AMPK levels (*P* = 0.0004 and *P* = 0.0014, respectively), indicating enhanced apoptosis and impaired AMPK activation (Figure 4b–f). In contrast, combination therapy with GMC1 and irisin resulted in a marked reduction in Bax (*P* = 0.0004), cleaved caspase‐3 (*P* = 0.0170) and p‐JNK (*P* = 0.0123) levels, while significantly increasing Bcl‐2 (*P* = 0.0053) and p‐AMPK (*P* = 0.0041) expression in comparison to the IR group. These findings suggest that combination therapy exerts anti‐apoptotic effects through the modulation of these key proteins (Figure 4b–f). However, the administration of the AMPK inhibitor (CC) alongside the combination therapy abolished its protective effects. Pretreatment with CC resulted in significantly higher levels of Bax (*P* = 0.0462), cleaved caspase‐3 (*P* = 0.0253) and p‐JNK (*P* = 0.0269) proteins, while reducing Bcl‐2 (*P* = 0.0272) and p‐AMPK (*P* = 0.0055) levels compared to the combination therapy group. These results suggest that AMPK activation is crucial for the anti‐apoptotic effects of the combination therapy (Figure 4b–f).

### 3.5 Combination therapy enhances mitochondrial function in diabetic IR‐injured livers, while AMPK inhibition abolishes this effect

**FIGURE 5:**
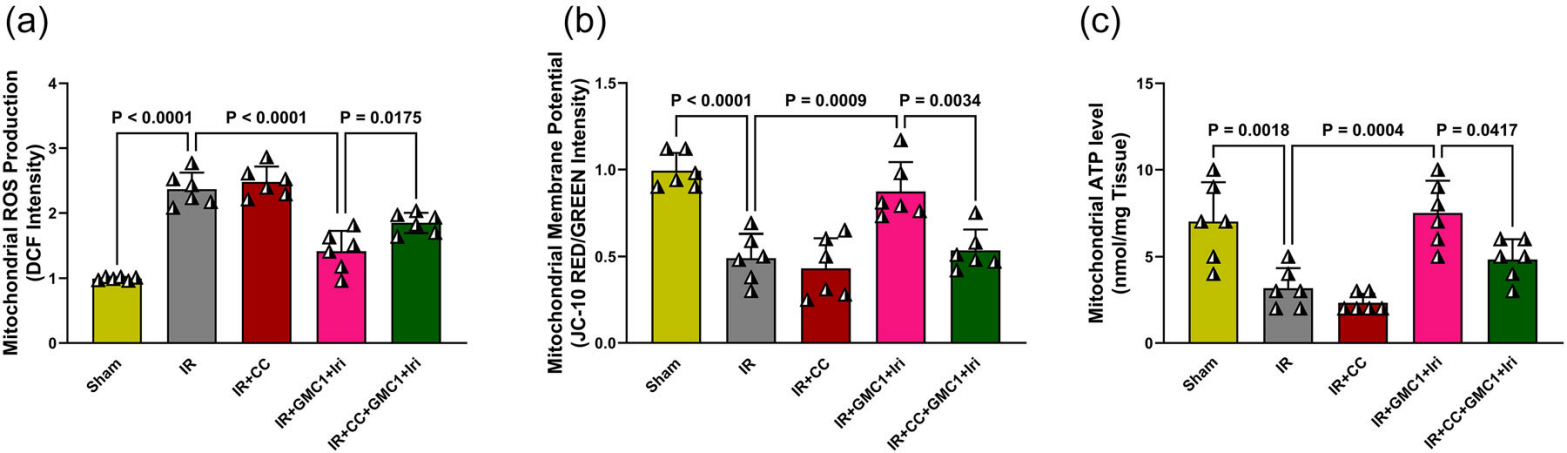
Combination therapy enhances mitochondrial function in diabetic IR‐injured livers, while AMPK inhibition abolishes this effect. Panels show mitochondrial reactive oxygen species (ROS) production (a), membrane potential (b), and ATP levels (c). Data are presented as means ± SD and analysed using one‐way ANOVA followed by Tukey's *post hoc* test (*n* = 6 per group). CC, compound C; DCF, 2′,7′‐dichlorodihydrofluorescein; GMC1, ginsenoside‐MC1; IR, ischaemia–reperfusion; Iri, irisin.

The aim of this study was to investigate mitochondrial apoptosis. Therefore, in addition to evaluating apoptotic markers, mitochondrial function was assessed. Furthermore, by using CC, we examined the role of the AMPK/JNK signalling pathway in mitochondrial apoptosis and the effects of combination therapy on this process. The IR group exhibited a significant increase in mitochondrial ROS production along with a marked decline in membrane potential and ATP levels compared to the Sham group (*P* < 0.0001, *P* < 0.0001 and *P* = 0.0018, respectively) (Figure 5a–c), indicating severe mitochondrial dysfunction. Combination therapy effectively mitigated these impairments, as evidenced by a significant reduction in mitochondrial ROS production and an increase in membrane potential and ATP levels compared to the IR group (*P* < 0.0001, *P* = 0.0009 and *P* = 0.0004, respectively) (Figure 5a–c). However, pretreatment with the AMPK inhibitor (CC) abolished these protective effects. Compared to the combination therapy group, CC administration resulted in a significant increase in mitochondrial ROS production (*P* = 0.0175) and a reduction in both membrane potential (*P* = 0.0034) and ATP levels (*P* = 0.0417), highlighting the essential role of AMPK activation in mediating mitochondrial protection (Figure 5a–c).

### 3.6 Combination therapy attenuates oxidative stress in diabetic IR‐injured livers, while AMPK inhibition abolishes this effect

**FIGURE 6:**
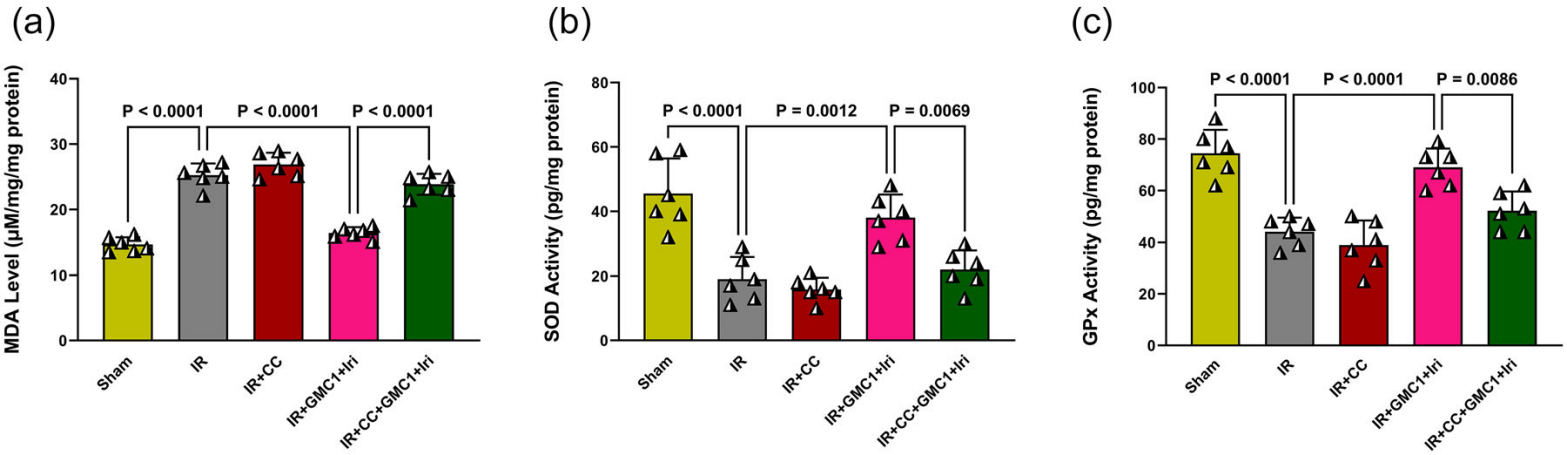
Combination therapy attenuates oxidative stress in diabetic IR‐injured livers, while AMPK inhibition abolishes this effect. Panels show the levels of malondialdehyde (MDA) (a) and the activities of superoxide dismutase (SOD) (b) and glutathione peroxidase (GPx) (c). Data are presented as means ± SD and analysed using one‐way ANOVA followed by Tukey's *post hoc* test (*n* = 6 per group). CC, compound C; GMC1, ginsenoside‐MC1; IR, ischaemia–reperfusion; Iri, irisin.

Oxidative stress plays a central role in IR injury, particularly in diabetic conditions, where excessive ROS production exacerbates cellular damage. To assess the impact of combination therapy on oxidative stress, we measured MDA levels as an indicator of lipid peroxidation, along with the activities of key antioxidant enzymes, SOD and GPx. As shown in Figure 6a–c, the IR group exhibited significantly elevated MDA levels and a marked reduction in SOD and GPx activities compared to the Sham group (*P* < 0.0001 for all), indicating severe oxidative damage. Combination therapy effectively mitigated oxidative stress by significantly lowering MDA levels and enhancing SOD and GPx activities compared to the IR group (*P* < 0.0001, *P* = 0.0012 and *P* < 0.0001, respectively) (Figure 6a–c). However, pretreatment with the AMPK inhibitor (CC) abolished these protective effects. Compared to the combination therapy group, CC administration resulted in significantly higher MDA levels (*P* < 0.0001) and decreased SOD (*P* = 0.0069) and GPx (*P* = 0.0086) activities, highlighting the crucial role of AMPK activation in modulating oxidative stress (Figure 6a–c).

## 4 DISCUSSION

This study aimed to evaluate the hepatoprotective effects of combined treatment with GMC1 and irisin in diabetic rats subjected to IR injury. Our findings indicate that pretreatment with these agents significantly improved liver function. Notably, combination therapy effectively reduced serum levels of AST, ALT and LDH while mitigating hepatic histopathological alterations. The observed hepatoprotection appears to be primarily associated with the suppression of mitochondrial apoptosis, leading to a subsequent reduction in oxidative stress. More importantly, our results suggest that these protective effects are mediated, at least in part, through activation of the AMPK/JNK signalling pathway, a crucial regulator of mitochondrial homeostasis and apoptosis. This pathway plays a pivotal role in maintaining cellular integrity under IR‐induced stress by promoting mitochondrial quality control and reducing apoptotic signalling.

Previous studies have demonstrated that GMC1 monotherapy and irisin monotherapy confer protective effects in various models of IR injury, primarily in non‐diabetic, healthy animals without comorbidities (Bi et al., 2019; H. Li et al., 2024). GMC1 has been shown to mitigate oxidative stress, enhance mitochondrial biogenesis and improve cellular energy metabolism in both cardiac and hepatic IR models (H. Wang, Tian, Tan, et al., 2022; Y. Zhang, Xu, & Yanqing Zhang, 2022). Similarly, irisin has been reported to preserve mitochondrial function, reduce apoptosis and attenuate oxidative damage across multiple experimental settings (Bi et al., 2019; Y. Wang et al., 2021). However, in the present study, monotherapy with either GMC1 or irisin failed to provide significant protection against IR‐induced hepatic injury under diabetic conditions. This discrepancy may be attributed to the complex diabetic microenvironment, which is known to exacerbate IR‐induced hepatic injury through several interrelated mechanisms (Lejay et al., 2016). Chronic hyperglycaemia, insulin resistance and dysregulated lipid metabolism in T2DM contribute to persistent oxidative stress and mitochondrial dysfunction, which not only elevate baseline cellular injury but also reduce the hepatoprotective efficacy of single‐agent therapies (Y. Zhang et al., 2016). Diabetes impairs mitochondrial quality control by disrupting mitophagy, decreasing ATP production, and promoting mitochondrial permeability transition pore opening, all of which sensitize hepatocytes to IR‐induced apoptosis (Behrends et al., 2010; Yue et al., 2015). In addition, diabetes induces a chronic low‐grade inflammatory state characterized by activation of the NOD‐, LRR‐ and pyrin domain‐containing protein 3 (NLRP3) inflammasome, further amplifying the inflammatory cascade during IR injury. This heightened pro‐inflammatory milieu can blunt the cytoprotective effects of individual pharmacological agents, thereby limiting their therapeutic effectiveness (Panés et al., 1996).

Our results indicate that combination therapy with GMC1 and irisin offers significantly enhanced hepatoprotection compared to either monotherapy. The superior efficacy of this dual approach likely stems from its ability to engage multiple protective mechanisms simultaneously. Rather than relying on a single signalling pathway, GMC1 and irisin may exert complementary or synergistic effects by targeting distinct but interconnected molecular networks (Patel & Saravolatz, 2006). In our study, combination therapy robustly activated the AMPK/JNK signalling axis, which plays a pivotal role in mitochondrial homeostasis, bioenergetic maintenance and apoptotic regulation. AMPK activation enhances mitochondrial resilience by increasing ATP synthesis and reducing oxidative stress, while JNK modulation contributes to apoptotic control and cellular adaptation to stress (Chen et al., 2018). This therapeutic strategy may exert protective effects not only through mitochondrial pathways but also via non‐mitochondrial mechanisms. Although our study did not directly assess these pathways, previous research suggests that AMPK‐dependent mitophagy can facilitate selective clearance of damaged mitochondria, reducing ROS production and apoptotic signalling (Cai et al., 2022). Additionally, inhibition of the NLRP3 inflammasome and pyroptosis may help attenuate inflammation‐induced hepatic injury (Jiménez‐Castro et al., 2019; Wu et al., 2022). These potential non‐mitochondrial protective mechanisms, inferred from earlier studies, require further investigation in future research to fully elucidate their roles. Due to the multimechanistic pathogenesis of hepatic IR damage in diabetes, combined therapy that targets mitochondrial and non‐mitochondrial mechanisms seems to be more effective against liver damage (Ahmadieh & Azar, 2014). Altogether, these data emphasize the weakness of monotherapy against diabetic IR damage and the superior quality of combination therapy. By modulation of several pathological drivers of liver injury, this strategy has the potential to improve hepatocyte function and survival in at‐risk patients, especially diabetic and metabolically abnormal ones. Further efforts are necessary to discover other protective pathways, maximize the treatment regimens, and translate these observations into translational and clinical contexts.

### 4.1 Limitations and suggestions

Despite the promising findings, several limitations should be acknowledged. First, the study was conducted exclusively on diabetic male rats, and potential sex‐specific differences in therapeutic response were not explored. Second, while key pathways related to mitochondrial function and apoptosis were investigated, other regulated cell death mechanisms – such as necroptosis and ferroptosis – were not comprehensively assessed. Third, although the combination therapy with GMC1 and irisin demonstrated efficacy in the short term, its long‐term safety, durability of benefit and translational potential require further evaluation. Moreover, the potential systemic effects of hepatic IR injury on remote organs such as the kidney and lung were not addressed in this study and should be investigated in future research.

### 4.2 Conclusion

In summary, the present study highlights the enhanced effectiveness of combination treatment of GMC1 and irisin over monotherapy in mitigating hepatic IR injury, especially in diabetic status. Through improvement in mitochondrial function, prevention of apoptosis, and minimizing oxidative stress, this therapy represents an encouraging treatment to preserve liver integrity from ischaemic stress insults. These results carry important clinical significance, particularly in candidates for liver transplantation, hepatic resectional surgery, and patients with trauma‐ or shock‐related hypoperfusion, situations in which IR damage is a prominent cause of postoperative morbidity. Patients with metabolic comorbidities like diabetes are of particular concern since they are even more vulnerable to IR‐induced liver injury due to pre‐existing mitochondrial dysfunction and compromised antioxidant defences. Thus, the development of this combination therapy as a preconditioning treatment may enhance hepatic tolerance, decrease postoperative morbidity and improve recovery outcomes in this high‐risk population. Future studies must address optimal dosing regimens and additional protective mechanisms, with translational research to establish effectiveness in the clinical environment. Finally, the inclusion of mitochondria‐targeted combination therapy in perioperative care regimens may prove to be a breakthrough in the enhancement of liver function and patient recovery in high‐risk surgical and critical care practice.

## AUTHOR CONTRIBUTIONS

Jie Lin and Lei Han were responsible for the overall design of the project. Jie Lin, Lei Han, Zhigang Ma, Bo Yuan, and Yabin Yu conducted the experimental work and collaborated in analysing and interpreting the results. Jie Lin and Lei Han assisted in drafting the manuscript. Bo Yuan and Yabin Yu supervised the whole project. All authors have read and approved the final version of this manuscript and agree to be accountable for all aspects of the work in ensuring that questions related to the accuracy or integrity of any part of the work are appropriately investigated and resolved. All persons designated as authors qualify for authorship, and all those who qualify for authorship are listed.

## CONFLICT OF INTEREST

None declared.

## Data Availability

The datasets utilized in this study can be obtained from the corresponding authors upon reasonable request.
